# The portrayal of gender in Marvel and Star Wars media targeted towards children

**DOI:** 10.3389/fsoc.2024.1338914

**Published:** 2024-02-15

**Authors:** Lucy Louise Clarke, Benjamin Hine

**Affiliations:** Department of Psychology, University of West London, London, United Kingdom

**Keywords:** Marvel, Star Wars, gender roles, thematic analysis, children’s media, masculinity, femininity

## Abstract

An abundance of previous research has investigated how gender has been portrayed within feature length films produced by Walt Disney Animation Studios, particularly those within the Disney princess franchise. However, the Disney corporation acquired the Marvel and Star Wars franchises in 2009 and 2012, respectively, which was likely a strategy for the corporation to obtain characters that would capture the imagination of boys and men. The current qualitative study explored how gender is portrayed by leading protagonists in these texts, utilising thematic analysis, which was necessary considering little is currently known in this domain. The researchers analysed series one of Avengers Assemble and series one of Star Wars Rebels. Interpretation of the data led to the development of several themes and subthemes based on the gendered portrayals within each series. Overall, the findings suggest that there was more overt gender stereotyping in Avengers Assemble when compared with Star Wars Rebels, meaning that the former could be particularly problematic for children who may replicate its messages. The current study has facilitated a greater understanding of the gendered messages that may be consumed by children who engage with Marvel and Star Wars media. Future research is needed to assess the relationship between such messages and children’s behaviour.

## 1 Introduction

The portrayal of gender in Disney princess animations has been widely examined (Towbin et al., 2004; Giroux and Pollock, 2010; England et al., 2011; Dundes and Streiff, 2016; Streiff and Dundes, 2017a,b; Hine et al., 2018; Primo, 2018). However, little research has investigated the portrayal of gender within the Marvel and Star Wars franchises, which Disney acquired in 2009 and 2012, respectively. This gap is problematic as Disney’s acquisition of these franchises was likely motivated by the corporation’s desire to own content that captures a male centric audience (Koushik and Reed, 2018; Wu, 2021) as boys reported greater personal interest in superheroes than classic prince characters (Dinella et al., 2017). Therefore, to understand the gendered messages that may be consumed particularly heavily by young boys, expanding analyses of the portrayal of gender to incorporate Disney’s newly acquired Marvel and Star Wars franchises is essential.

Although very little research has investigated the portrayal of gender within Marvel and Star Wars media, previous studies have examined the gendered portrayals within superhero media more broadly. Such research finds that the portrayal of both male and female superheroes were largely in line with broader gender role stereotypes (Miller et al., 2016). For example, male superheroes were highly muscular, powerful, and more violent while female superheroes were more sexualised and helpless (Miller et al., 2016). Female superheroes are also significantly outnumbered by males (Baker and Raney, 2007; Miller et al., 2016). Additionally, violence and aggression tended to be perpetrated more by protagonists than antagonists, and more by male characters than female characters (Muller et al., 2020). This is supported by Miller et al. (2016) who found that males utilised weapons and fighting skills more frequently than females, meaning that they were portrayed as more aggressive and violent overall.

Similarly, Baker and Raney (2007) suggested that although male and female superheroes seemed to display elements of stereotypical masculinity, females were more emotional, more attractive, more worried, and more likely to be excited in a crisis than males (Baker and Raney, 2007). Alternatively, males were more likely to express anger and be portrayed as threatening than females (Baker and Raney, 2007), whereas females were more likely to have a mentor, and more likely to work in a group, suggesting some adherence to gender norms in superhero narratives. Further, Marvel superheroes “when acting in their capacity as a hero… talked about their emotions, accepted physical comfort, and expressed trust significantly less often than when acting in their capacity as self” (Shawcroft and Coyne, 2022, p. 232) suggesting superheroes do not express vulnerability.

Taken together, these findings suggest that stereotypical masculinity is favoured in superhero content. Indeed, it has been said that superhero narratives seem to “indulge in fantasies about the heroes’ unlimited ability to protect a silent and largely feminized humanity from that which threatens it” (Stabile, 2009, p. 87). However, further research is needed to investigate whether the portrayal of gender within Marvel content specifically is in line with superhero media more broadly.

Further, it is possible to view the masculinity presented in superhero media as hegemonic. Hegemonic masculinity refers to a masculinity that is dominant over others, making it relational (Donaldson, 1993; Connell, 2005; Connell and Messerschmidt, 2005; Messerschmidt, 2019). It is perhaps the power associated with domination that makes “heroism… so tightly bound into the construct of hegemonic masculinity” (Connell, 2005, p. 234), which means that it may be an important concept in understanding the portrayal of gender in superhero narratives that focus largely on superheroes protecting less powerful characters (Stabile, 2009; Kort-Butler, 2013). Hegemonic masculinity has been associated with traits such as being “unemotional, independent, non-nurturing, aggressive, and dispassionate—which are seen as the causes of criminal behavior” (Connell and Messerschmidt, 2005, p. 840), as well as some more positive ones, such as financially providing and being sexually active (Connell and Messerschmidt, 2005). Because hegemonic masculinity is a relational concept, femininity can be defined as its inversion (Connell, 2005). Therefore, femininity may be defined as weakness, passivity, being nurturing and having emotional tendencies. Additionally, hegemonic masculinity has been associated with economic and “public” work while femininity has been associated with domestic spaces and largely devalued domestic and “reproductive work” (Kreimer, 2004, p. 22; Connell, 2005; Elgarte, 2008). Because superheroes are likely to be dominant over others, it is possible that such representations of masculinity and femininity will be prevalent in superhero narratives. Conversely, the portrayal of gender within the Star Wars franchise has been even more scarcely studied. However, the Star Wars story world is largely deemed male dominated and patriarchal, with the utilisation of The Force for example, being reserved for male characters in the original film trilogy (Pianka, 2013; Bruin-Mole, 2017). For Bettis and Sternod (2009), it seems as though a large part of acquiring the power to utilise The Force and become a Jedi is learning to suppress emotion which could be rooted in the expectations of stereotypical masculinity (Parent et al., 2019) and disguised as a source of power. Therefore, the patriarchal culture that the Star Wars galaxy may emulate could represent a depiction of men and women that may be concerning.

However, more recently released Star Wars media has arguably portrayed a more progressive depiction of women with its leading protagonist Rey (Bruin-Mole, 2017). This has led researchers to state that the Star Wars franchise’s depiction of women reflects the developments within the women’s movement in the US (Bruin-Mole, 2017; Langsdale, 2019; McGucken, 2020). This means that examining the depiction of male and female protagonists within some of the franchise’s most recently released animated content is important as this is likely to facilitate an understanding of the messages that are being consumed by children engaging with it.

Further, the relationship between the messages in Marvel and Star Wars media and children’s gendered behaviour is largely unknown. Although not directly relating to the Marvel franchise, Coyne et al. (2014) found that superhero media predicts masculine behaviour in pre-school aged boys in the US, but not girls. Further, superhero media engagement was also predictive of higher levels of weapon play for both boys and girls (Coyne et al., 2014). These findings suggest that although girls may not replicate the masculine behaviour of superheroes in the same way that boys do, both boys and girls may be more likely to use toy weapons, or use objects as weapons in their play scenarios, because of such media. This is potentially concerning, as “weapon play” may be associated with levels of aggression displayed by children (Watson and Peng, 1992, as cited by Coyne et al., 2014, p. 426). Further, this adds to the “[n]early 3,000 studies and reviews [which] have found a significant relationship between media violence and real-life aggression” (Strasburger, 2009, p. 655), including in videogames, according to a recent metanalysis (Prescott et al., 2018), suggesting a real-world impact of this messaging.

Moreover, when boys wore Marvel superhero outfits, they were less likely to show feminine-typed toy preferences and prosocial behaviour than when they were in gender-neutral costumes or feminine-typed outfits (Coyne et al., 2021). Additionally, engagement with superhero media in children aged between four and five years old was “associated with endorsement of the muscular [body] ideal and some aspects of hegemonic masculinity five years later” (Coyne et al., 2022, p. 642). For example, engagement with superhero media in a sample of children with the mean age of 4.83 years was related to superhero engagement at wave two of data collection when the mean age of the sample was 10.05 years. The later engagement predicted lower egalitarian attitudes towards men and women. Taken together, these findings suggests that boys associate and emulate the masculine gender stereotyped behaviour associated with Marvel superheroes when they play as these characters (Coyne et al., 2021) and that superhero narratives inform attitudes towards men and women more broadly (Coyne et al., 2022). These findings suggest that there is a relationship between the messages that children are consuming from superhero media and the behaviours they display. However, little is known about the relationship between Star Wars engagement and children’s behaviour.

Overall, little is known about the portrayal of gender in Marvel and Star Wars media, despite there being some evidence that there is a relationship between the messages in superhero media and children’s gendered behaviour (Coyne et al., 2014, 2021, 2022). Therefore, the current study aimed to qualitatively analyse the representation of male and female leading characters in Marvel and Star Wars media that is targeted towards children. Because this study was a precursor to research that analysed the relationship between children’s engagement with Marvel and Star Wars media and their behaviour which utilised a sample of children aged between 4 and 11 years, it was important that the series analysed were suitable for the majority of the sample. As a result, content that was rated as suitable for children aged 6 and above was analysed. This is also the lowest age rating for Star Wars and Marvel media on Disney+ United Kingdom, the streaming service which was utilised for data collection in the current study. The study was guided by two research questions:

How is gender portrayed within a Marvel animated television series?

How is gender portrayed within a Star Wars animated television series?

## 2 Method

### 2.1 Materials

The researchers selected one animated series from the Marvel and Star Wars franchises suitable for viewers aged six and above. Because the Avengers live action films had the highest grossing figures of all the Marvel films, indicative of the characters’ popularity, the first series of Avengers Assemble (2012–2019) was analysed as those same characters featured in that series. Series one, which was released in 2012, consisted of twenty-six episodes, each approximately twenty-four minutes long (10.4 h of content). Additionally, series one of Star Wars Rebels (2014–2018) was selected as it was released in 2014 which was closest in time to the release of the first series of Avengers Assemble (2012–2019) than any other Star Wars animated series comparatively. There were fifteen episodes within the first series of Star Wars Rebels (2014–2018), each being approximately 24 min long, equating to six hours of content. The first series of Avengers Assemble (2012–2019) and Star Wars Rebels (2014–2018) were analysed so that the narratives could be understood by the researcher, as well as considering that if individuals searched either of these series on streaming services (such as Disney+), they would be presented with the episodes in chronological order. Data was collected from leading male and female characters only. This approach enabled an in-depth understanding of the portrayal of female and male characters that were likely to be the most narratively important and influential to the audience. This decision was also important for practical reasons, namely, the time involved in data collection. Disney+, Disney’s online streaming service was utilised to access each series.

### 2.2 Procedure

The procedure of data collection in the current study mirrored that established by Towbin et al. (2004) in which Disney animated feature length films had been thematically analysed. Each episode was watched once in its entirety before the researcher rewatched the episode and began identifying codable units/segments of data. A unit of data was any content that the researcher deemed to be relevant to the research question. For example, a unit of data could be extracted from a scene in which the male Avengers were sat around a table discussing an attack plan while Black Widow (the only female Avenger) was stood behind them with very little verbal input. This image would suggest that the male Avengers were more active in strategic planning and Widow’s input was less valued. Moreover, her physical position would suggest she is on the periphery of the team. If imagery was captured as a data unit, a description would be written, and the description would then be coded. While imagery is important to capture the perhaps more subtle and implicit gendered messages, speech between the characters could also be captured as units of data. If the data unit was speech, it was transcribed exactly, utilising subtitles for accuracy. When all the data from series one of Avengers Assemble (2012–2019) and Star Wars Rebels (2014–2018) was collected, it was then analysed in a “bottom-up” data driven thematic analysis as defined by Braun and Clarke (2006, 2013). The data collected from the Marvel and Star Wars franchises were treated as separate data sets. Each unit of data was coded. A code was a brief word or short sentence that captured why the unit of material was interesting and relevant to the research question (Braun and Clarke, 2006). Once provisional themes had been established for each data set, they were discussed with the second author before the final themes were defined and named. Although inter-rater reliability measures were not implemented in the study, a pilot study was conducted in which both authors identified data units and coded part of the first episode of series two of Avengers Assemble (2012–2019). A high rate of agreement was established in coding of the units that were identified, which suggested validity and reliability in the process of data collection before the target material was analysed. The pilot study was also useful as it enabled the primary coder to identify where data units could be split into multiple units to obtain more detail in the gendered portrayals, as the second coder identified more data units in their analysis. However, of the data units that were identified by both authors, the majority (approximately 80%) were coded with similar themes.

## 3 Results

### 3.1 Avengers Assemble (2012–2019)

The male Avengers protagonists analysed were Iron Man, Captain America, Thor, Hulk, Hawkeye and Falcon. The female Avenger analysed was Black Widow. The current research aimed to answer the research question: How is gender portrayed within a Marvel animated television series? The main themes established were *stereotypical masculinity/lad culture* and *being flawed and fallible*. Each of the main themes had several subthemes. The themes and subthemes generated from Avengers Assemble (2012–2019) can be found in Table 1.

**Table 1:** Themes and sub-themes answering the research question: “How is gender portrayed in a Marvel animated television series?”

Theme	Subtheme	Data unit
Stereotypical masculinity/lad culture	Teamwork	Thor fights Attuma—knocks him with his hammer towards Hulk who punches him, sending him flying up into the sky (E16, unit 14)
	Battles and aggression	Iron Man: ‘Looks like we get to smash the cabal ahead of schedule’ (E21, unit 32).
	Banter	Hawkeye: ‘So, are you guys gonna hug now, or what?’ (E13, unit 89)
	Odd one out (Widow)	Falcon: ‘This must be Widow’s room. No way it’s Hulk’s. Looks like my mum cleaned it’ (E3, unit 46).
	Strength and muscularity	[NA. This is visibly portrayed continuously, not within specific data units]
Being flawed/fallible	Respect is earned	Iron Man: ‘That an acceptable plan Mr. Hawkeye?’Hawkeye: ‘I’ll let you know when it works’ (E1, unit 96)
	Anger needs to be controlled	Iron Man: ‘Thanks to Mr. Anger-Management, we have an unknown number of unstable particles loose in the tower’ (E23, unit 47)
	Vulnerable without ‘suits’	They are being dragged into the whirlpool. Iron Man: ‘Steve, I’m sorry. My armor, it’s not enough’ (E13, unit 81)

#### 3.1.1 Main theme: stereotypical masculinity/lad culture

There were many concepts of stereotypical masculinity and “lad culture” (Phipps and Young, 2015) endorsed by the male protagonists within Avengers Assemble (2012–2019), and between the Avengers and the male villains that they fought. Additionally, the masculinity that was portrayed in the series aligns with hegemonic masculinity. Interestingly, much of this was also endorsed by Widow, the only female protagonist within the series. This suggests that conforming to stereotypically masculine gender norms is essential to be a valued member of the Avengers team. There are four subthemes within the *stereotypical masculinity/lad culture* main theme that will be explored below.

##### 3.1.1.1 Subtheme: teamwork

There were many incidences of teamwork within each episode of the Avengers Assemble (2012–2019) particularly during battles where the team almost always worked together to overcome an antagonist or enemy. The teamwork presented was similar to the teamwork that is portrayed in sports dominated by men, one that “celebrates and promotes toughness, competitiveness, violence, and confrontation” (Kessler et al., 1982, p. 5 as cited by Yang-Huang et al., 2017, p. 325) and such traits are also considered hegemonic.

For example, in the units below, several members of the team worked together to attack a villain named MODOK who had combined with the Adaptoid, creating a particularly dangerous villain:

Widow, Falcon and Captain America all jump and land ready.

Widow jumps and kicks MODOK.

Falcon lands and stomps on head.

Captain America hits him with shield.

Captain America hits him with shield again.

Widow and Falcon shoot him with their weapons.

Widow tumbles/ forward rolls to get out the way of MODOK’s blast (Episode 8, units 84–90).

The combined aggression displayed by several members of the team throughout these units reflects that stereotypically masculine behaviour was fundamental to the teamwork that was presented in the series. The Avengers’ unity was often celebrated for creating a formidable force for fighting evil. For example, a villain stated that:

‘It is said that the together, the Avengers can face threats that no single hero can’ (Episode 13, unit 15),

which provides evidence of their power—a stereotypically masculine concept—being associated with their ability to work together. Further, the power that the Avengers have is both over the villains that they defeat and the society that they work to protect. There are scenes throughout the series where the team are admired by civilians as though they are celebrities which makes this narrative more pronounced. It is perhaps the admiration and power experienced by the team that makes the masculinity represented by the Avengers appear to be tied in with the concepts of stereotypical and hegemonic masculinity (Donaldson, 1993; Connell, 2005; Connell and Messerschmidt, 2005; Messerschmidt, 2019)

##### 3.1.1.2 Subtheme: battles and aggression

A very significant proportion of the series was dedicated to the Avengers fighting villains/antagonists. In these battles the Avengers utilise weapons as well as direct aggression (such as a punch or a kick). Because every episode had a battle, and every battle lasted several minutes (sometimes almost the entire episode) the amount of violence and aggression displayed was vast. The Avengers had a light-hearted approach to their battles and often framed them as a source of entertainment. Therefore, both the prominence of aggression, and the casual way it was presented, could be reasons for concern. For example, when Iron Man articulates a plan to take down Doomstroyer, a villain featured in episode 10, there were the following reactions:

[Hulk:] ‘Stupid plan. Sounds fun’ (Episode 10, unit 103).

Battles seemed to be welcomed and celebrated in other episodes:

[Iron Man:] ‘Mind if I crash the party? I’m running a scan on your playmate Thor. One second and I should know exactly how we should deal with…’ (E4, unit 7).

Iron Man refers to getting involved in a battle as a party, implying that it is a fun pastime, rather than a potentially life-threatening pursuit.

Additionally, the Avengers would have physical altercations with each other, sometimes solely for fun. For example, in episode two—*The Avengers Protocol Part 2*, Hulk and Thor decide to battle:

Hulk and Thor go into the training room and reminisce about old battles.

They start to battle for ‘old time’s sake’.

[Hulk:] ‘You kept thinking you could knock me down’.

[Thor:] ‘Did more than think, I think’.

[Hulk]: ‘Wouldn’t want to mess up that pretty hairdo’. [The battle starts]. They both smile (episode 2, units 68–76).

The reminiscent tone in which Hulk and Thor discussed their battles implied they have previously enjoyed, and will miss, their opportunities to fight. Further, the fact that they smile during their battle shows that fighting is a form of entertainment and perhaps, of male bonding.

##### 3.1.1.3 Subtheme: banter

Consistent with the ‘lad culture’ of the Avengers, banter was the main source of communication between the teammates (Phipps and Young, 2015) as they almost constantly mocked each other. In a typical example of an interaction below, Iron Man wants to update Captain America’s armor, providing him with more technology to improve his battle skills:

[Iron Man]: ‘Hence, I took your boring old butt-kickers and teched them up a bit.’

[Captain America]: ‘More tech does not always equal more better.’

[Falcon]: ‘I do not think that’s proper grammar.’

[Captain America]: ‘I’m dumbing it down for the genius.’

[Iron Man] turns on the boots so Cap starts flying in the air.

[Iron Man] laughs as Cap is being flung around and hitting things….

[Iron Man]: This, my friend is what technology was made for’.

(E17, units 4–14).

Throughout these units, there is constant tone of Captain America and Iron Man mocking each other, including being entertained by physically hurting one another.

Additionally, genuine sincerity was rare between Avengers, and it was often followed by mocking, or aggression, as if to balance the less stereotypically masculine form of communication. For example, in episode eighteen *Mojoworld*, Hawkeye accidently breaks one of Hulk’s much-loved glass sculptures. Throughout the episode Hawkeye refuses to apologise in a genuine way. They are then forced to work together and at the end of the episode, they have the following interaction:

[Hulk to Hawkeye]: ‘Already apologized’ [not even looking at him].

[Hawkeye]: I know… just wanted it to be sincere’ [Looking at him]

[This interaction is shot from behind so you can see the side of Hawkeye and only the back of Hulk]. Hulk: ‘We’re good’ [Elbows him hard so he goes flying].

Hawkeye returns to his room to find several boxes of pickles and believes they are a gift from Hulk. He starts opening the boxes and they are all empty. Hulk has left a note that reads ‘I.O.U. 15 boxes of pickles. Got hungry’ and HE is angry and screams: ‘HULK!’ (E18, units 126–128).

Two Avengers are sharing a sincere moment in the units above that becomes overshadowed by their utilisation of banter and mocking of each other. There was also a significant amount of banter between the Avengers and other secondary characters such as the villains that the team fought.

##### 3.1.1.4 Subtheme: odd one out (Widow)

The subtheme *odd one out (Widow)* speaks to the notion that although Widow emulated *stereotypical masculinity/lad culture,* she was also portrayed as somewhat different to the male Avengers. She was absent for nine episodes out of the twenty-six within the series. Importantly, when she was absent, it was rarely mentioned by other characters suggesting that rather than being a vital part of the team, she was a disposable addition to it.

Episode eight, *Molecule Kid,* is one of the few episodes in which Widow is at the forefront. In the episode, Widow attempts (unsuccessfully) to lead herself and Hawkeye through a mission while keeping it hidden from the other Avengers. In this episode there are several instances of sexism. For example, Hawkeye overtly implies that being a leader is masculine by referring to Widow as sir:

‘Permission to fire the champion shot you should’ve let me fire back in the alley, sir!’ (E8, unit 34).

Just a few second later, Hawkeye says:

‘Where did you learn to drive, huh, video games?’ (E8, unit 38),

drawing upon a well-known sexist stereotype that women are inadequate drivers. Further, the following interaction is another way in which Widow’s gender is mocked:

[They end up in honey].

Widow: ‘Honey?

Hawkeye: ‘Yes, dear?’

Widow: ‘No, we are stuck in honey.’

Hawkeye: ‘You said the wand does not do organics. So, this is not honey, honey. Hey great mission plan so far by the way. Perfection. Really.’ (E8, unit 62).

Hawkeye repeatedly calls Widow honey, a remark/nickname that is often adopted by romantic partners. Therefore, heteronormative jokes are made between men and women, and these were less likely to be utilised in an interaction between two male Avengers.

Overall, there were numerous ways in which Widow was presented to be both a part of the Avengers’ ‘lad culture’, but also as the odd one out.

It is also noteworthy that there are very few secondary female characters present throughout the series. Interestingly, however, in Episode 23, *One Little Thing*, Falcon’s mother is present. She is a source of stress from the start of the episode as Falcon believes she is unaware that he is an Avenger and would not approve of his role. Because of this, she is presented as overprotective and nurturing throughout the episode, the latter evidenced by her bringing home-baked cookies for Falcon and his teammates. However, at the end of the episode she reveals she knew that Falcon was an Avenger and has therefore been underestimated by her son. Overall, the representation of her being a nurturing female figure seems significant considering there are so few female characters throughout the series. It may also situate Widow as the *odd one out* more strongly as she presents more traits associated with *stereotypical masculinity/lad culture* than Falcon’s mother.

##### 3.1.1.5 Subtheme: strength and muscularity

The male Avengers are all physically muscular. The majority of them wear skin-tight superhero suits through which their muscle definition, particularly of their chest, arms, and shoulders, are clearly visible. Hulk wears ripped trousers, meaning his muscular torso is always on display. In contrast, Iron Man’s suit is a hard red shell. However, it is designed in the shape of a muscular body, with particularly defined shoulders, arms, and chest, making his physicality like that of the other male Avengers. The physical representation of the male Avengers matched their physical performance as they displayed extreme strength throughout the series. This could suggest that the skin-tight suits some of the male Avengers wore, which are revealing of their bodies, is justified because their bodies are seen as active (Strong, 2003).

Although Widow also wears a skin-tight suit, her physical representation is noticeably different from the male Avengers. She has no muscle definition whatsoever, with her shoulder width and biceps being noticeably smaller and less defined than those of the male Avengers. In some scenes, she appears to be around half the width of her male counterparts which has implications for power dynamics that are associated with muscular bodies over slimmer ones. Her slim body could be a sign of her being sexualised. It should be acknowledged that she has large breasts which are exaggerated by her small waist. Her slimmer more sexualised frame is more representative of a stereotypically feminine body that is more passive despite her physical performance largely being the opposite, something that is discussed in relation to female body builders who will often pose differently to their male counterparts in competitions (Strong, 2003).

#### 3.1.2 Main theme: being flawed and fallible

The second main theme represents that although the Avengers are an incredibly powerful team and are successful in overthrowing each of the villains they encounter in the series, they must work hard to do so, with it sometimes seeming as though they are close to being defeated (and inevitably, this near defeat motif adds to be drama and appeal of these narratives). Additionally, the masculinity presented by the male protagonists throughout the series is seen to have some considerable drawbacks. The concept of *being flawed and fallible* will be explored within each of its subthemes: *respect is earned, vulnerable without suits* and *anger needs to be controlled.* These subthemes can be related to the concept of “manhood” needing to be proven (Vandello and Bosson, 2013) as well as some of the “undesirable traits” (Connell and Messerschmidt, 2005, p. 840) associated with hegemonic masculinity which will be explored throughout this section.

##### 3.1.2.1 Subtheme: respect is earned

The concept of *respect is earned* was most frequently portrayed by Falcon, the newest and youngest Avenger. At the start of episode three, Falcon moves into the Avengers tower. Iron Man reveals his room to him, which is full of equipment and hardly usable. However, as the episode unfolds, Falcon then manages to save the Avengers, almost singlehandedly. At the end of the episode Iron Man says:

‘I’ll have the stuff moved out’ [of your room].

[Falcon]: ‘After today, you are building me a bigger room. Off the helipad. You can afford it’ (E3, unit 131),

implying that he has managed to earn the respect of the leader.

Further, the *respect is earned* subtheme is also represented by Iron Man. He is the leader of the Avengers but is frequently challenged by his teammates. For example, in Episode 21—*The Numbers*, the following interactions happen during a group training session which Iron Man is leading.

Falcon: ‘Is there a point to this?’

Captain America: ‘I’m with Falcon. This is not the best team building exercise you have ever come up with’.

Hulk [Has a ball in his hand and pops it]: ‘What he said’.

Iron Man: [Describes that he has come up with a new system and algorithms since the Cabal hacked him]. ‘The Stark probability engine. It predicts a sure path to victory in any combat situation.’

Captain America: ‘There are no sure things in combat. You’re forgetting the human factor’.

Iron Man: ‘The human factor is in there, it’s just insignificant. Statistically speaking’.

Captain America: ‘Insignificant? [cracks muscles] Do it again. We’ve got a little surprise of our own’ (E21, units 13 to 19).

The units above provide an example of how Iron Man must continue to earn the respect of his teammates. Several of them firstly express doubts over the efficiency of the training exercise he is leading, and Captain America then questions the logic of his invention. This suggests that respect is not gained easily within the Avengers team.

The concept respect being earned rather than naturally granted is similar to the concept of ‘manhood’ being a status that must be achieved (Vandello and Bosson, 2013), perhaps because masculinity is tied to social power and the patriarchal gender order (Connell, 2005). Therefore, “manhood” is seen as a social accomplishment that also needs to be maintained as it is possible to lose. The way in which the Avengers do not offer their respect to one another by default, seems to tie into this view of masculinity.

##### 3.1.2.2 Subtheme: vulnerable without suits

Iron Man and other male Avengers appear to be vulnerable underneath the protection of superhero suits, a concept that could represent the notion of males feeling as though they need to keep their vulnerabilities hidden. The first episode of Avengers Assemble (2012–2019) begins with Iron Man reuniting the team because his suit has become damaged and he needs the help of the Avengers to rescue Captain America, who was captured by a villain (Red Skull). Red Skull explicitly states the source of Iron Man’s power is his suit:

‘You’re just a fool in a machine, Stark! Without your technology you have nothing.’ [There are cracks in his suit and there are sparks coming from it, as if to reassert Red Skull’s point. RS goes on]: ‘No instinct for battle, no fire to lead. You hide behind armor so you do not have to make sacrifices for victory!’ [Jarvis says, ‘power is at 20% sir, and you have a new problem teleporting in]’ (it’s MODOK). (E1, unit 20)

Red Skull is clearly attempting to belittle Iron Man in the unit above, and undermine his skill and power—essentially, emasculating him.

Throughout episode 13, Captain America and Iron Man enter a ship occupied by villains by disguising themselves meaning that neither of them have their usual suits or the associated weapons. Although this does not seem to trouble Captain America, Iron Man repeatedly asserts that they need their armor back. Iron Man goes on to reveal that he feels insecure and vulnerable without the protection of his suit:

Captain America: ‘Thanks, but…’ Iron Man: ‘But with my armor gone, all I do is improvise. Steve, even without your shield, you are still Captain America. Without my armor, I’m just…’ (E13, unit 58).

This suggests that he feels inadequate, providing more evidence for the suit being a symbol of masculinity for Iron Man to hide his insecurities behind.

##### 3.1.2.3 Subtheme: anger needs to be controlled

The *anger needs to be controlled* subtheme particularly related to Hulk. Hulk is the focus of three episodes in the series, and in two of these his anger is portrayed as a problem. This is also a recurring theme across many other episodes of the series. The following interactions happen at the start of episode nine, where the Avengers are responding to a monster attack:

[Hulk]: ‘Less talking, more smashing’ [has damaged the arms of the chair by gripping them].

[Falcon]: ‘Speaking of rampaging monsters…’ […]

[Captain America]: ‘Let him out before he breaks down the door again.’

[Hulk roars and kicks down the door. He jumps out and lands on a gigantic beast, clearly having no fear of it. He punches beast].

[Iron Man and Thor jump out of plane to follow Hulk.] Iron Man: Don’t you just love it when the Hulk goes all rage monster? Like we need another one to deal with.’

Thor: [Laughs] ‘Hopefully, his rage will serve us well in battle this time’ Iron Man: ‘Famous last words’ (E9, units 4–13)

Throughout these units, it is clear that Hulk’s anger has been continuously problematic. Both Iron Man and Captain America’s comments imply that his anger is detrimental, rather than of use. Later in the episode, Hulk’s strength and control of his rage ultimately enables the Avengers to overcome the villain for which he is rewarded:

Iron Man: ‘Hulk got his rage under control and saved the entire city. That’s why he’s Avenger of the month’ (E9, units 149 to 161).

Therefore, within one episode Hulk’s anger has gone from being the team’s main issue to being the source of the resolution *only when it is controlled and channeled appropriately.*

Anger seems to be portrayed as natural yet problematic when it leads to mindless destruction throughout the series However, controlled anger facilitates the team’s success meaning that a difficult balance between utilising anger to help the team, rather than hinder it needs to be maintained.

**Figure 1:**
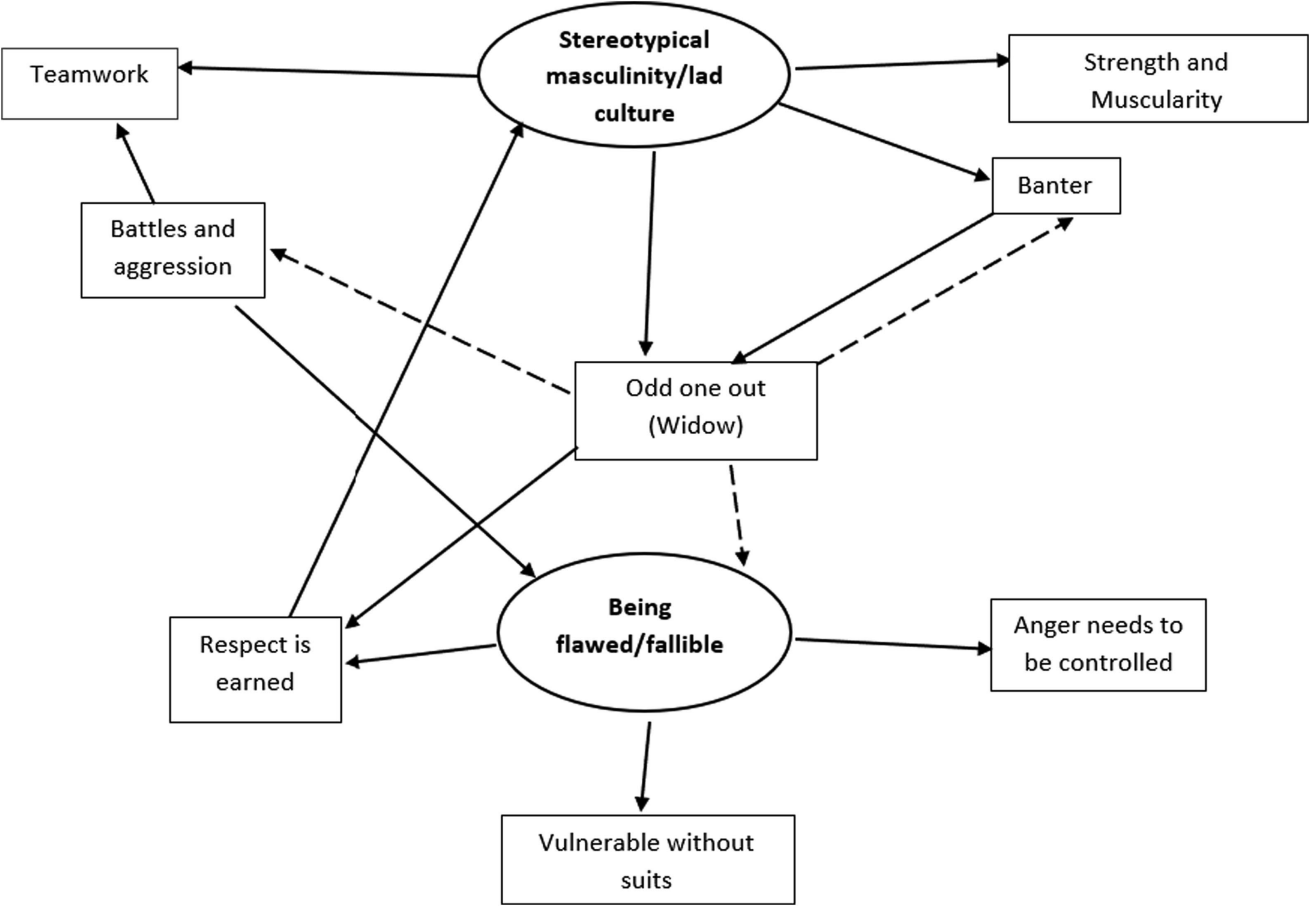
Thematic map: how is gender portrayed in a Marvel animated television series?

Figure 1 illustrates the final thematic map that answers the research question, how is gender portrayed in a Marvel animated television series? It intends to show the connections between each of the themes (in ellipses) and subthemes (in boxes). The solid arrows indicate a causal relationship whereas the dotted arrows represent conflicts.

### 3.2 Star Wars Rebels (2014–2018)

The characters analysed from Star Wars Rebels (2014–2018) were the crew members of the ship, the *Ghost.* Kanan, Ezra and Zeb were male protagonists and crew members, and Hera and Sabine were female protagonists and crew members. Three themes were established to answer the research question: How is gender portrayed in a *Star Wars* animated television series? The themes established were *multifaceted masculinities*, *strong female figures* and *gender(ed) co-operation* (see Table 2).

**Table 2:** The themes and subthemes established to answer the research question: ‘How is gender portrayed in a Star Wars animated television series?’

Theme	Sub-theme	Unit of data
Multifaceted masculinities	Toxic/stereotypical masculinity	Hera: ‘Zeb. The pig! Scare it’ Z: ‘What? How?’ H: Just be *you*.’ (E11, unit 53)
	Mentor/leader	Kanan: ‘We’ll draw them away! Get spectre-2 and Trayvis to the hatch’ (E12, unit 78)
	Mentee	Kanan: ‘Stunts like that put us all in jeopardy. That is exactly why you need Master Luminara to teach you discipline’ (E5, unit 60)
	Emotional vulnerability	Kanan: ‘Your emotions clouded the vision. It takes…’ (E12, unit 108)
Strong female figures	Leader/mother figure	Hera: ‘Positions, everyone. We’re going in’ (E14, unit 86)
	Action girl	Hera: ‘Sabine, man the nose gun!’ (E2, unit 21)
Gender(ed) co-operation	Hierarchies	Hera: ‘So, how’s the Jedi training going with Kanan?’ Ezra: ‘Jedi training? Never heard of it.’ Hera: ‘We’ll see about that’ (E3, unit 51)
	Heteronormative tones	Hera: ‘You’re welcome, dear’ [they hug]’ (E15, unit 113).

#### 3.2.1 Main theme: multifaceted masculinities

The main theme *multifaceted masculinities* reflects the portrayal of the male protagonists in the series (namely, Kanan, Ezra and Zeb), with each subtheme capturing a distinct aspect of the masculinity reflected in the series.

##### 3.2.1.1 Subtheme: toxic/stereotypical masculinity

*Toxic/stereotypical masculinity* was represented most clearly and consistently by Zeb. Zeb was frequently aggressive and seemed to enjoy being aggressive more than his counterparts. He was also unwilling to express emotions outside of anger. For example, in episode two—*Spark of Rebellion Part 2*, Ezra was captured by the enemy and Zeb leaves him on their ship:

[Zeb] Looks sad/hopeless as he returns to the ship without Ezra. Slumps on to the floor (E2, unit 25).

However, when he had to tell the rest of his crew what happened, he seemed to be unwilling to express the remorse that he displayed when he was alone moments earlier. He had an angry and defensive tone and implied that Ezra had no importance to him or the crew:

[Zeb]: ‘Oh, come on. We were dumping him after the mission anyway! This saves us fuel. They’ll go easy on him. He’s just a kid’ (E2, unit 34).

Similarly, in episode eight—*Empire Day,* Ezra shows signs of being unwilling to express his emotions. In the episode, the crew plan and execute an attack on the Empire and it is clear that Ezra is upset but refuses to discuss it. For example, when he takes the crew to his family home that has since been abandoned (and it is unclear as to whether Ezra’s parents are alive), the following interaction ensues:

[Kanan]: ‘You were coming here today. This was your home, wasn’t it? Where you grew up?’

[Ezra]: ‘I grew up on the streets, alone.’ (E8, units 39 and 40).

The units above provide evidence of Ezra being emotionally guarded and unwilling to be truthful about a potentially traumatic situation with his crewmates.

Overall, Zeb seemed to represent stereotypical and toxic masculinity most strongly, although this was also seen in Ezra.

##### 3.2.1.2 Subtheme: mentor/leader

Kanan, an adult male character, Ezra’s Jedi master and is responsible for his training. He is often assertive towards Ezra, giving him orders regularly. This is shown in episode ten—*Path of the Jedi* when Ezra has missed training:

[Ezra]: ‘Hey Kanan. Sorry I’m late. I was with Sabine. So, are you gonna invite me in?’

[Kanan]: ‘You did not knock, so what makes you think you need an invite?’

[Ezra]: ‘I’m sorry.’

[Kanan]: ‘Then you should knock first.’

[Ezra]: ‘Not for that. For missing training.’

[Kanan]: [angrily] ‘It’s all the same thing. The fact that you do not see it [sighs]. Ezra when we were on that asteroid you made a dangerous connection through the Force. Now I have to know if you are ready.’

(E10, unit 3).

Kanan is clearly in a mentoring role where he is attempting to teach Ezra the skills he needs and test him with challenges.

Additionally, Kanan had a role as leader of the crew. He would often direct the crew when they were completing missions on foot (rather than on the ship, where Hera, a female character and pilot, was more in control). Therefore, both Kanan’s roles in the crew, as a mentor and a leader, are positions in which he has power as a knowledgeable and experienced male, suggesting this is a fundamental part of his character and masculinity. Interestingly though, his role as the leader of the crew also means that he is vulnerable. Later, in the same episode Kanan is battling with the Inquisitor (a villain within the series) and has no way of escaping:

[Kanan] Pinned against the wall—in the air.

[Kanan]: ‘Spectre-2, get out of here’

[Hera]: ‘Not an option, Kanan.

[Kanan]: ‘No time! Go!’

[Ezra]: ‘We cannot!’

[Kanan]: Hera!

[Hera looks sad and presses a button] (E13, unit 93).

Kanan has demanded that Hera leave him to keep the crew safe, sacrificing his own life. In the following episode, he is shown to be captured and tortured by Imperials, and thus, vulnerable rather than powerful (as he is within the crew). However, because Kanan has demanded to be left in one last assertion directed towards Hera, his masculinity could be perceived to remain intact as he is in control and decisive, as well as sacrificial, brave, and protective of the rest of his crew.

##### 3.2.1.3 Subtheme: mentee

Ezra is the focus of much of series one of the series due to his growing skills and utilisation of the Force. He is presented as continuously learning, particularly from Kanan. Because of this, Ezra’s role within the crew and team is very much as a mentee.

When Ezra and Kanan must face a battle with the Inquisitor, Ezra has to protect Kanan who is injured:

[Inquisitor]: ‘Ah, yes, good. Go on. Unleash your anger [laughs] I will teach you what your master could not’.

[Ezra]: ‘You do not have anything to teach me.’

[Inquisitor]: ‘The darkness is too strong for you, orphan. It is swallowing you up, even now.’ (E9, unit 55).

In the unit above Ezra is deemed to be vulnerable because of his lack of complete training.

In non-battle situations, Ezra is seen to create problems for the crew as he needs to learn key skills. He has gone against orders before the following interaction with Kanan:

[Kanan]: ‘Stunts like that put us all in jeopardy. That is exactly why you need Master Luminara to teach you discipline’.

[Ezra]: ‘I was just trying to follow your example’

[Kanan]: ‘Try following the plan instead’ (E5, units 60 to 62).

Overall, although Ezra has a natural ability to access and utilise the Force which is celebrated in much of the series, he is represented as a mentee—a young male in training. It is perhaps no coincidence that the oldest male is the person who is responsible for Ezra’s training, suggesting that the skills of the Force are both masculine and age related, arguably representing the notion of ‘manhood’ being earned (Vandello and Bosson, 2013).

##### 3.2.1.4 Subtheme: emotional vulnerability

A concept that further linked Ezra and Kanan’s masculinity was emotional vulnerability. Although Kanan is in control of Ezra’s training, he frequently expresses self-doubt. For example, in episode ten—*Path of the Jedi*, Kanan sets Ezra a challenge. However, it transpires that he doubts his own abilities as a mentor.

[Kanan]: ‘It’s true. I’m not sure of my decision to train Ezra. Not because of him or his abilities, because of me, because of who I am’.

[Kanan]: ‘…I feel his abilities are growing faster than I can teach him’ (E10, units 58 and 59).

Insecurity and emotional vulnerability are therefore shown by a male character who has power over his mentee and the rest of the crew, suggesting his masculinity is multifaceted.

Additionally, during Jedi training, there were some sincere and emotionally charged conversations between Ezra and Kanan such as in the example below:

[Ezra]: ‘My parents spoke out and I lost them, and I do not… [angrily grunts] I do not want to lose you guys, okay? Not over this.’

[Kanan]: ‘Hey. All of us have lost things. And we will take more losses before this over. But we cannot let that stop us from taking risks. We have to move forward. And when the time comes, we have to be ready to sacrifice for something bigger.’

[Ezra]: ‘That sounds good, but it’s not so easy.’

[Kanan]: ‘It’s not easy for me either’ (E13, units 45 to 49).

When Kanan and Ezra are alone, Ezra discusses his emotional vulnerabilities, such as his feelings of inadequacy and fear of loss. Therefore, private conversations seem to allow for emotional vulnerability between Ezra and Kanan, perhaps because being emotionally vulnerable is part of their utilisation of the Force, and, more broadly, in their fight as rebels.

#### 3.2.2 Main theme: strong female figures

The second main theme was established based on the representations of the female protagonists, Hera and Sabine, in the series. Similarly to the *multifaceted masculinities* theme, by presenting two leading female characters, it seemed that there were two possible representations of the female gender role, each represented by a subtheme: *leader/mother figure* and *action girl*.

Interestingly, in the final episode of the series, Fulcrum whom Kanan and Hera had been relying on for intel regarding missions they could complete to earn the crew credits (their currency), is revealed to be a female character, named Ahsoka Tano. Throughout the series Kanan and Hera had been secretive about the source of their intel, and when Sabine questioned them, she had assumed Fulcrum was male. Although the representation of Falcrum/ Ashoka Tano was beyond the scope of this work, it is noteworthy that she was a strong, rebellious female with knowledge and power, suggesting that her representation would fit within the *strong female figures* subtheme.

##### 3.2.2.1 Subtheme: leader/mother figure

Hera is represented as a leader, mainly within the domain of the crew’s ship, which she owns and pilots. Her role as a pilot and leader is invaluable and fundamentally important to the crew’s pursuits. For example, episode nine opens with the crew in danger and Hera making several demands of them:

[Hera]: ‘Sabine, I need you in the nose gun, now!’ (E9, unit 3).

[Hera]: ‘Ezra, Nav-computer is off-line. With Chopper down, I need you to fix it’

[Ezra]: ‘Not exactly my specialty’

[Hera]: ‘Well, make it your specialty and make it fast. Or this ship becomes a real ghost’ (E9, unit 10).

Hera is presented as taking control in adversity in the units above. However, Hera’s role as a leader is almost exclusively confined to the ship, which is also the crew’s home, making it a domestic space. That Hera is in control of the domestic space has implications for the gendered narrative within the series, as such spaces have been historically marked as feminine domains (Connell, 2005). Also in-line with broader gender role stereotypes, she is portrayed to be a mother figure in the series, often handling sibling-like issues that arise between crew members. For example, in episode four, Ezra and Zeb are arguing and Hera shouts:

[Hera]: ‘Enough. This is my ship you are wrecking, and I want you off it. [Gives them a market list]. Do not even think about coming back without at least one meiloorun fruit’ (E4, units 21 to 23).

Overall, Hera’s role as a leader is fundamentally important to the crew. She is skilled and necessary to the missions However, her role as a leader within the crew had tones of mothering suggesting her role was partially defined by stereotypical gender expectations also.

##### 3.2.2.2 Subtheme: action girl

Sabine’s role in the crew is as a tough and action-based girl. Like Ezra, Sabine is in her teens and is important in missions and battles when they happen both in the air (from the ship), as well as on foot. This means that when Hera is flying the *Ghost* during the on-foot missions and battles, Sabine provides female representation. In such scenarios she is confident, skilled (especially with explosives) and aggressive.

For example, episode fifteen—*Fire Across the Galaxy* opens with Sabine attacking stormtroopers:

[Sabine]: ‘Miss me bucketheads?’

Jumps and climbs a wall.

They’re shooting at her. Sabine: ‘Yep, you definitely missed me’ [Jumps down from the roof].

[Ezra and Zeb] Are waiting in the wings.

[Ezra]: ‘Sabine’s distraction is working’ [jumps, pulls herself up on things].

(E15, units 1 to 13)

In the units above, Sabine is clearly a natural action girl displaying agility, strength, confidence, and skill.

#### 3.2.3 Main theme: gender(ed) co-operation

The *gender(ed) co-operation* theme represents that the team are successful in most of their missions and battles, and often use teamwork in order to complete them. Each member of the mixed-gender crew had important and valued roles. However, there were some *hierarchies* represented in the team, some of which were gendered and will be explored below. Additionally, there were some *heteronormative tones* within the series. These concepts are represented as sub-themes and will be discussed in turn.

##### 3.2.3.1 Subtheme: hierarchies

While Kanan and Hera are both presented as leaders, there are signs of gendered domains to their leadership. Hera leads on the ship, which is partly a domestic space, and is seen as a motherly figure, while Kanan is a leader in battles, a more masculine environment. Despite this, Hera and Kanan were both presented as more powerful than the rest of the crew when it came to decision-making and survival. The crew needed to complete missions to obtain credits (their currency) for food and supplies. Hera and Kanan often shut down conversations when the other crew members questioned the source of their intel regarding such missions, highlighting their power. This is shown in episode seven—*Out of Darkness:*

[Kanan]: ‘It’s Hera’s job to find missions that create problems for the Empire and profit for us. If she trusts the contact, I trust the contact. No questions asked’ (E7, units 24 and 25).

There was a second hierarchy within the crew. Because Ezra’s Jedi training is much of the focus of the series, his development was seen as vitally important while Zeb and Sabine’s roles were less focused upon. Ezra was the most important of the (non-leader) crew members which also elevated Kanan’s status because he was Ezra’s mentor. It also notable that Ezra and Kanan, two male characters, are the only crew members to have the ability to utilise the Force meaning that they battle the most powerful villains in the series (The Inquisitor and Lord Vader). Kanan and Ezra then are arguably shown to be the most important and valued crew members by the villains who wish to maintain the hold of the Empire—both of whom are males, suggesting a gendered representation of skill and power.

##### 3.2.3.2 Subtheme: heteronormative tones

The crew can be seen as a (non-biological) family with Hera and Kanan as the parental figures (McGucken, 2020) which represents a heteronormative dynamic. There were subtle insinuations that Kanan and Hera were in a romantic partnership. Hera, on a few occasions refers to Kanan with romantic terms:

[Kanan]: ‘Can we discuss this later?’

[Hera]: ‘That’s fine, love. But we will discuss it’ (E3, unit 52).

Her use of the word ‘love’ seems particularly interesting considering it may be utilised to soften her assertive tone (often associated with masculinity) within the sentence.

Additionally, in the last episode of the series when the crew have successfully rescued Kanan he expresses his gratitude, and she responds:

‘You’re welcome, dear’ [they hug]’ (E15, unit 113).

Again, it is Hera who utilised a romantic term (and Kanan never reciprocates).

Additionally, heteronormativity is shown through interactions between Ezra and Sabine. In the first episode Sabine takes off her helmet and Ezra sees her for the first time. He looks momentarily amazed and a few seconds later uses a flirtatious tone while he says:

‘My name’s Ezra, what’s yours’ (E1, unit 78).

Ezra speaks to Sabine in this flirtatious tone in other episodes throughout the series, despite her showing no romantic interest in him.

Overall, this subtheme represents that although the crew consists of males and females who successfully work together as a team, there are heteronormative tones present. This both allows and challenges the reading of a heteronormative family dynamic being portrayed. Perhaps then, Star Wars Rebels (2014–2018) implies that heteronormative romance is inevitable in a mixed-gendered team.

**Figure 2:**
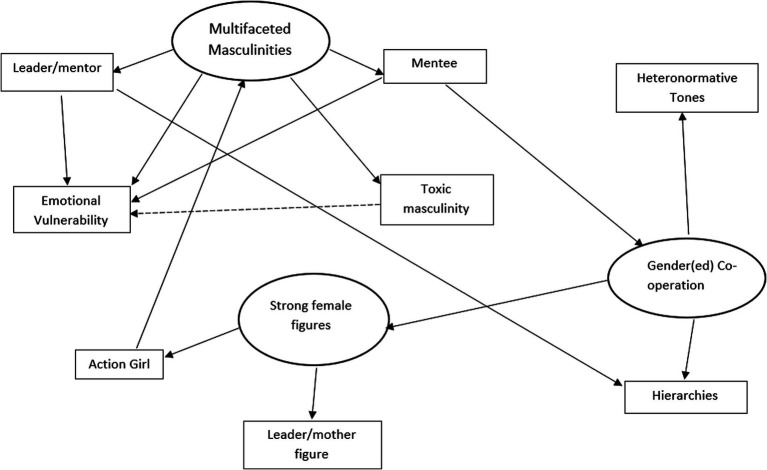
Thematic map: how is gender portrayed in a Star Wars animated televisions series?

Figure 2 illustrates the final thematic map that answers the research question, how is gender portrayed in a Star Wars animated television series? It intends to show the connections between each of the themes (in ellipses) and subthemes (in boxes). The solid arrows indicate causal relationships whereas the dotted arrows represent conflict.

## 4 Discussion

This study was the first to investigate the depiction of gender within Marvel and Star Wars content that is rated as age appropriate for children, namely, one series of Avengers Assemble (2012–2019) and one series of Star Wars Rebels (2014–2018). This was important because engagement with superhero media statistically predicted stereotypically masculine behavioural profiles in young boys, and higher rates of weapon play (Coyne et al., 2014). Therefore, understanding the portrayal of gender in two popular and influential franchises that have each been acquired by the Walt Disney corporation was essential if this is likely to be influencing the young children engaging with it. Now that Marvel and Star Wars media are owned by the Disney corporation, it is important to consider the differences and similarities in the gendered portrayals within these franchises.

### 4.1 Comparing the portrayal of male characters

Firstly, it is important to acknowledge that the current study found stereotypical aspects of masculinity in both Avengers Assemble (2012–2019) and Star Wars Rebels (2014–2018). However, the stereotypical masculinity seemed more exaggerated and narratively dominant in Avengers Assemble (2012–2019) possibly because as a group, the Avengers were powerful and admired, traits that are associated with hegemonic masculinity (Connell, 2005). The Avengers seemed to be hegemonic in that they had more power over the largely subordinate human society (as suggested by Stabile, 2009), whereas the crew in Star Wars Rebels (2014–2018) were fighting against the dictatorial government body ruling over them (the Empire). As a result, the latter were more subordinate. For example, the crew had to take jobs to gain credits that they could exchange for essential supplies whereas the Avengers (and particularly Iron Man) were extremely wealthy and lived in relative luxury. Therefore, it could be argued then that the larger role of emotional vulnerability in Star Wars Rebels (2014–2018) when compared with Avengers Assemble (2012–2019) could reflect that their masculinity was less defined by associations with hegemonic masculinity.

In Star Wars Rebels (2014–2018) Ezra and Kanan were portrayed to reflect on their fears and vulnerabilities in order to utilise the Force. Therefore, emotional vulnerability was associated with power in the series. Bettis and Sternod (2009) suggested that love is discouraged within the Star Wars films. However, although not necessarily a reflection of love in the romantic sense, it is through Ezra’s attachments to his crew members that his skills are able to develop. Indeed, Kanan explicitly tells Ezra that “you have to let your guard down. You have to be willing to attach to others.” This suggests that in Star Wars Rebels (2014–2018), perhaps rather than suppress their emotions and minimise their connections to others, males are encouraged to do the opposite, and are rewarded with the power of The Force for doing so. Therefore, the message in Star Wars Rebels (2014–2018) may be teaching young boys that stoicism (Wall and Kristjanson, 2005; Parent et al., 2019), is not the only component of masculinity that leads to success.

In contrast, emotionally charged conversations were rarely displayed by the Avengers, and when they were, the characters were wearing their civilian dress rather that their superhero suits. In this way, superhero suits could be interpreted as a metaphor for masculinity, beneath which individuals are more able to express emotions and vulnerabilities, concepts that are rarely expressed by men who feel pressured to adhere to stereotypical gender norms (Parent et al., 2019). This also supports the work of Shawcroft and Coyne (2022) who found that superheroes were less able to show vulnerabilities as heroes (in their suits) than as humans (without their suits) suggesting that being emotionally vulnerable is simply not ‘super’.

Additionally, uncontrolled anger was presented as a significant and consistent issue within Avengers Assemble (2012–2019) which is notable due to a lack of displays of other emotions. Uncontrolled anger often led to unnecessary destruction. However, there were several occasions where being able to control anger was celebrated. The implications for children exposed to the positive response to controlled anger in such media are unclear. The message could be that although uncontrolled anger is an inconvenience, acting on such emotion is ‘natural’ for men, which could normalise problematic behaviour.

A further difference between the Avengers superheroes and the crew of Star Wars Rebels (2014–2018) was that the Avengers engaged with each other almost exclusively through banter. Banter has been regarded as a feature of ‘lad culture’ which has been associated with sexual harassment and the mistreatment of women (Phipps and Young, 2015). Although unsurprisingly, sexual harassment was not portrayed in Avengers Assemble (2012–2019) it is possible that young boys being exposed to the concepts of ‘lad culture’ from a young age may be more likely to normalise and accept it later in life, when it may be related to such issues. In this way, the series could have sexist undertones in line with the (mis)treatment of women associated with ‘lad culture’ (Phipps and Young, 2015; Nichols, 2018). Although the use of banter may create a sense of problematic “lad culture”, it can also be prosocial (Bergin et al., 2003; Huuki et al., 2010) and important in developing relationships. Indeed, there was evidence of this in Avengers Assemble (2012–2019) as even though the characters constantly mocked and belittled each other, it was almost always perceived to be in good jest and facilitated bonding. The use of banter between the characters may also be narratively important—friction between teammates is common in “team films” as it adds interest to the group dynamic (Strong, 2013).

Lastly, Avengers Assemble (2012–2019) seemed to suggest that respect needed to be earned through masculine endeavours such as being skilled in battles, utilising intellect, and proving your worth within the team. This relates to the concept that ‘manhood’ is earned and proven via social achievements, personality, and behaviours (Vandello and Bosson, 2013). Because “manhood” is achieved, it can also be lost meaning that “men’s behaviors (particularly stereotypically masculine behaviors) are often motivated by *an ongoing need to prove manhood status to others*” (Kimmel, 1997, as cited by Vandello and Bosson, 2013, p. 103, emphasis added). This also links to the concept of manhood being earned through public action (Vandello and Bosson, 2013). Further,

“the precariousness of manhood, for example, can explain why men: value status and achievement; display traits such as assertiveness and dominance; engage in risky and aggressive behaviors; avoid femininity in their appearance, personality, and conduct; and experience anxiety and stress when they fail to achieve cultural standards of masculinity” (Vandello and Bosson, 2013, p. 107).

Arguably then, much of the stereotypically masculine behaviours displayed by the male Avengers could be explained by their continuous need to prove and maintain their status as heroes. This is similar to the portrayal of the hierarchy that is present between Ezra and Kanan and a mentee and mentor dynamic—the older male character was more skilled than the younger male.

### 4.2 Comparing the portrayal of female characters

The key difference in the portrayal of female protagonists between Avengers Assemble (2012–2019) and Star Wars Rebels (2014–2018) found in the current study was that in the latter, the female protagonists seemed to be more genuinely valued. For example, Hera’s piloting skills were vital to the team, and she was presented as a leader alongside Kanan. In comparison, Widow, the only female Avenger, seemed to be an add-on to the team and did not seem as genuinely valued. The Avengers worked without her just as successfully for a large proportion of the series even though she portrays stereotypical masculinity. Baker and Raney (2007) also found that female superheroes seemed to be stereotypically masculine, which seemed to suggest that masculinity was conducive to being super. Despite Widow’s performance of masculinity, she was *still largely on the periphery of the team*. This indicates that engaging in masculinity is not enough for female superheroes to fully ‘fit in’. Additionally, the lack of female representation in Avengers Assemble (2012–2019) is in line with previous research which finds that male superheroes are much more prevalent than female superheroes (Baker and Raney, 2007; Miller et al., 2016).

There is some subtle stereotyping of female characters in Star Wars Rebels (2014–2018) also. The leader/mother-figure subtheme described Hera’s role within the crew—she is portrayed as a leader almost exclusively within a domestic space suggesting that Hera is still somewhat constrained by feminine gender role stereotypes. Historically, the private sphere has been equated with femininity and women, whereas the public sphere has been equated with masculinity and men (Kreimer, 2004; Connell, 2005; Elgarte, 2008). This gendered distinction of the public and private spheres facilitated the subordination of women (Kreimer, 2004; Connell, 2005; Elgarte, 2008), which makes this representation of Hera in Star Wars Rebels (2014–2018) problematic.

Further, Hera’s role as a leader within the private sphere is contrasted with Kanan’s role in mentoring and passing on practical skills that could be utilised for important public work– namely, the taking down of the Empire. Therefore, a gendered private and public distinction can be identified in Star Wars Rebels (2014–2018), when comparing the roles of Hera and Kanan. Hera was also presented as a mother figure to the younger crew member which associated her with nurturing/reproductive work and the private sphere (Elgarte, 2008) more strongly. This implied that for a female figure to be a leader which is contrary to gender stereotypes, she must also be motherly—supporting gender stereotypes. This is perhaps, as McGucken (2020) suggested, representative of fourth wave feminist sensibilities whereby Hera and Sabine:

“certainly meet fourth wave criteria, they also reflect traditional female roles and suggest an inherent tension in defining female, femininity, and feminism itself. This reflects divisions and important factors in identifying the fourth wave, which is marked by a multiplicity of definitions and contradictions—and a welcoming of them in order to better understand the next steps within the feminist project” (McGucken, 2020, p. 155).

Additionally, neither of the female crew members could utilise the Force, which was also true in the original Star Wars film trilogy. This is a particularly interesting narrative choice considering the sequel trilogy, which was released just one year after the first series of Star Wars Rebels (2014–2018), had a female Jedi as its leading protagonist which was considered a more empowering representation of women than those in the earlier trilogies (Bruin-Mole, 2017; McGucken, 2020). That the power to utilise the Force was represented to be an exclusively male phenomenon in Star Wars Rebels (2014–2018) was therefore more consistent with the original trilogy. This could suggest that Star Wars was not willing to commit to representing female characters as powerful in this way, perhaps because it was still relatively unclear how its fanbase would respond to such a change.

Overall, this may suggest that Star Wars Rebels (2014–2018) seems to provide children with more examples of how men and women can successfully work together and respect one another than Avengers Assemble (2012–2019).

### 4.3 Limitations and future directions

The current thematic analysis was conducted on the first series of Avengers Assemble (2012–2019) and Star Wars Rebels (2014–2018). Therefore, it is possible that the portrayal of men and women would have developed throughout subsequent series of these television shows and analysing all available episodes may have led to a greater understanding of the portrayal of gender within them. However, there were four series of Star Wars Rebels (2014–2018), each with a minimum of fifteen episodes, and four series of Avengers Assemble (2012–2019) each with a minimum of twenty-three episodes. Due to time constraints, it was not possible to analyse all episodes. Future research could investigate the portrayal of gender throughout the entirety of an animated series associated with Marvel and Star Wars franchises, to mitigate for this limitation.

Further, because the current research focused on one series of animated content from the Marvel and Star Wars franchises, the themes are not generalisable to those franchises more broadly. For example, there are many other animated series associated with both franchises, such as Star Wars: The Clone Wars (2008–2020) and The Avengers: Earth’s mightiest heroes! (2010–2012), as well as many feature length films. Such content should be analysed in future research and the findings compared with those of the current study to develop a more complete understanding of the portrayal of gender within these franchises.

Thirdly, the method adopted in the current study largely followed Towbin et al.’s (2004) whereby media was viewed by the researcher and when a unit of analysis was identified, a description of the content was written. If the unit contained speech, it was copied verbatim utilising the subtitle function of Disney+. Codes, themes, and subthemes were then established based on the written units of analysis for each series, rather than the units as they appeared in the television series directly. This is a potential limitation as the units of analysis may not represent the content as accurately as intended. However, the researcher ensured that important detail was contained within the descriptions of the data units to mitigate for this. Additionally, although there were no reliability checks conducted on the target data, a pilot study was conducted whereby consistency in identifying units was considered and discussed by the authors. Therefore, the pilot study provided evidence that the data units and codes being identified were consistent.

### 4.4 Implications

The current study could be utilised to discuss the portrayal of gender within the Marvel and Star Wars franchises with parents and teachers of children who are likely to engage with such media. Three main findings could be communicated. Firstly, parents and educators could be made aware of the more ‘positive’ portrayal of male characters engaging in emotionally charged conversations in Star Wars Rebels (2014–2018) in comparison to the lack of sincere conversations and emotional expression in Avengers Assemble (2012–2019). Based on this, parents could be advised to balance some of the “negative” representations of male protagonists largely suppressing emotions in Avengers Assemble (2012–2019) with those in Star Wars Rebels (2014–2018) as it is likely that viewing a more balanced portrayal of emotional expression would be beneficial to young viewers.

Secondly, media violence is impactful on children’s aggression (Strasburger, 2009; Prescott et al., 2018) therefore, the pervasiveness of violence in Marvel and Star Wars media found in this study should be communicated. Additionally, the relationship between aggression in superhero media and children’s use of toy weapons in their play has also been documented (Coyne et al., 2014) arguably making the violence in such narratives a particular cause for concern. Importantly though, further research investigating the relationships between engagement with these particular franchises and children’s behaviours is warranted. It is possible that because the Avengers seem to particularly celebrate aggression and are in a position of elevated social status, often linked with hegemonic masculinity (Connell and Messerschmidt, 2005), Marvel media will have a larger impact on children’s levels of weapon play and aggression than Star Wars media. However, whether this is the case should be investigated to provide additional support and weight to the information that has been gained in the current study when communicating findings to parents, educators, and children themselves.

Thirdly, the subtleties of gendered messaging in children’s media should be communicated to parents and educators. The current study suggested that it is possible that the portrayal of women in Star Wars Rebels (2014–2018) could be considered ‘positive’ and ‘progressive’. However, when considering the nuances in those portrayals, it is possible to see some problematic gendered messages. By raising awareness of the subtleties of gendered representations in children’s media, parents and educators may be able to make more informed decisions about the media their children consume. It would also be necessary to make caregivers and educators aware that previous research has found that children’s engagement with superhero media predicted higher agreement with statements associated with males being superior to females, and a desire for a muscular body (Coyne et al., 2022). Therefore, because the current study has found that the muscular body is prevalent in these narratives, and the masculinity presented particularly within Marvel media can be seen to be hegemonic, such narratives are likely to have a relationship with their children’s behaviour and conceptuliastions of gender, according to previous research. Overall, it is important for caregivers to be aware of the sometimes-subtle gendered messages that are portrayed by Marvel and Star Wars media, and the potential impact this may have on their children’s behaviour.

## 5 Conclusion

This study, to the best of the researcher’s knowledge, was the first to qualitatively assess how gender is portrayed in Marvel and Star Wars media targeted towards children. This was essential given that the Disney corporation acquired these franchises, presumably, to target a young male audience more successfully. The findings suggested that there was a more overt portrayal of stereotypical masculinity in Avengers Assemble (2012–2019) in comparison to Star Wars Rebels (2014–2018). Additionally, the women in the latter series seemed to be more valued than the one female protagonist in Avengers Assemble (2012–2019) however, the female protagonists were stereotyped in both series. Overall, the current research suggests that there was more overt gender stereotyping in Avengers Assemble (2012–2019) which could suggest that this media may be particularly problematic for children engaging with it, who may replicate its messages (Bussey and Bandura, 1999; Coyne et al., 2014). Overall, the current study has facilitated a greater understanding of the gendered messages that may be consumed by children who engage with Marvel and Star Wars media. The findings also provide important background for investigating the relationship between children’s engagement with the Marvel and Star Wars franchises and their gendered behaviours, which should be investigated in future research.

## Data availability statement

The raw data supporting the conclusions of this article will be made available by the authors, without undue reservation.

## Author contributions

LC: Conceptualization, Data curation, Formal analysis, Investigation, Methodology, Project administration, Supervision, Validation, Visualization, Writing – original draft, Writing – review & editing. BH: Conceptualization, Methodology, Validation, Visualization, Writing – review & editing.
